# Solution to the Case for Diagnosis

**Published:** 1908-02-15

**Authors:** 


### SOLUTION TO THE CASE FOR DIAGNOSIS.

The subsequent course of the case for diagnosis, whose history up to the time of consultation has been given on page 496 of our last issue, was as follows:—

The day after the consultation, after a restless night, the right arm was found to be partially paralysed in addition to the complete paralysis of left arm, left intercostal and abdominal muscles, and left leg. The patient was just able to flex and extend the fingers of her right hand slowly and feebly. The power of the sphincters, previously good, was now lost, and the fæces and urine were passed involuntarily.

On examining the respiratory movements, it was found that the action of the diaphragm on inspiration was so feeble that its descent could hardly be noticed. No contraction could be detected in the intercostal muscles of the left side, and the very slight action of that side of the chest seemed to depend on the movements of the right side, whose intercostal muscles contracted well. The vesicular murmur in the right lung was distinctly more audible than was that in the left, especially in front. The heart's sounds were quite normal. The pulse rate was 120, the respiration rate 32.

Next day the girl appeared to suffer more pain in the neck, and the least movement increased it so much as to make her cry out, albeit feebly. Sensation was more impaired in the left arm and leg, and she complained of a want of power over the right leg. The pulse rate was 112, and the respiration rate 32.

On the third day after the consultation the paralysis continued to increase. It was complete down the whole of the left side below the neck, and of the right arm. Voluntary power over the right leg was rapidly failing; the toes could just be moved, and the muscles of the calf could be contracted, but not sufficiently to move the foot. No reflexes were obtainable. Respiratory movement seemed to be performed exclusively by the right side, and feebly by the diaphragm. The patient still complained of great pain in the neck, especially on the least movement. The pulse rate was 120, and the respiration rate 32. A few minutes later there was a sudden change for the worse. The girl abruptly became speechless, with livid face and purple lips, efforts at respiration being in gasps at intervals of twenty seconds. The only muscle that could be observed acting was the sterno-mastoid of the right side. There was no perceptible motion of the ribs on either side, and no abdominal movement. The heart sounds remained clear, and the pulse was full, beating at the rate of 90 per minute. The cardiac action gradually became slower and more feeble, and in twenty minutes the beating and sounds of the heart had become inaudible. Some short, gasping efforts at respiration were perceptible for a minute or two afterwards, and then the patient died.

---

In regard to the diagnosis in this case, there was at first a strong temptation to attribute the paresis to a functional origin. The case is a very good example of the extreme danger of lightly diagnosing hysterical paralysis—a point we have laid stress upon in a former article. It is far worse a mistake to diagnose as functional that which is organic and serious, than to incline to the view that a lesion is organic when it is really functional.

The case had, at first, very much the appearance of hysterical paralysis, at the time when the left arm was first affected and the left leg but partially. Here was a case of hemiparesis, in a girl of sixteen whose menstrual functions were in abeyance; who suffered from vertical headaches of great severity, similar to clavus; who dragged one leg, moving it with a sweep such as is so often seen in functional cases; no paresis of the face, and no apparent cause for any of the commoner forms of hemiplegia; no syphilis to cause thrombosis, no heart disease to cause embolism, no renal mischief to cause hæmorrhage, no vomiting and no optic nuritis to suggest tumour of the brain, no otitis media, injury, or lung trouble as a source for cerebral abscess. The wryneck and the cervical pain were not necessarily against a diagnosis of hysterical paralysis, but the sense of fullness in the deeper tissues of the upper part of the neck was the first point upon which stress was laid in diagnosing organic mischief.

The next point was the fact that sensation was impaired uniformly throughout the paretic limb: in hysteria the distribution of cutaneous insensibility is usually erratic; in the leg, for example, there may be complete anæsthesia from ankle to knee, without any of the foot or thigh—the area of anæsthesia not corresponding either to peripheral nerve distribution or to spinal segment representation. Moreover, hysterical anæsthesia is usually either complete or not present at all. In this patient anæsthesia was partial, and it corresponded to the distribution of motor paresis; it was, therefore, upon the face of it, organic in origin.

The third point which led to the diagnosis of organic mischief was the paralysis of the intercostal and abdominal muscles of one side, and the deficient action of the diaphragm, with evidence of one side of it acting less well than the other. This unilateral paralysis of the muscles of the trunk is probably never functional. Moreover, it is probably never due to a lesion of the cerebral cortex of one side, or in one internal capsule, such as might otherwise have been the cause of the hemiplegia. Paresis of the trunk muscles is possible from bilateral cerebral softening, in much the same way that similar bilateral softening may simulate bulbar paralysis; but unilateral paralysis of the trunk muscles practically excludes a cerebral lesion, and points to organic mischief on one side of the upper part of the spinal cord. It thus became possible, not only to decide that the lesion was organic, but also to locate it to the left side of the upper part of the spinal cord.

It is very rare for a lesion within the cord itself to cause hemiparesis; lesions within the cord mostly cause bilateral paralysis, except in the case of infantile palsy. The case, however, could not be one of that kind, because the trouble, instead of being acute in onset, worst at its beginning, progressing towards recovery, and unassociated with anæsthesia, was just the reverse—gradual in onset, slight at first, becoming progressively worse, and anæsthesia was present. It seemed probable, therefore, that there was something forming outside the upper part of the spinal cord and compressing it on its left side, finally interfering with it so much that the right side was interfered with also. Whether the "something" was spinal caries, or a new growth, it was scarcely possible to say.

---

The *post-mortem* showed that a small amount of disease is capable of producing dire results. The disease consisted chiefly in an enlargement of the odontoid process of the second cervical vertebra. This extended backwards, wearing through the dura mater. The new growth was a chondro-fibroma, possibly to a very mild extent malignant in character, which compressed and flattened the spinal cord on the left side of the median fissure. The compression of the cord was so great that it seemed as if a large portion of the nervous matter had been pushed from the left to the right side and partly upwards. The cord was swollen both above and to the right of the compressed part. The nervous matter on the left side was completely pulped; that on the right side less so.

No other disease was found in any part of the body. It was plain, therefore, that the left-sided hemiplegia, without implication of the face, was caused by the compression of the left half of the spinal cord just below the decussation of the pyramids, and that the paralysis of the left side, and finally of the right, became more complete as the tumour grew and further compressed the cord.

The softening extended for some distance below the actual seat of the tumour; hence the paralysis of the diaphragm and of the respiratory movements, first on the left side, and finally on both sides.

The sudden termination of the case was explainable as occurring when, the lower respiratory centre in the left side of the cord being already paralysed, extension of the softening on the right side finally reached the lower respiratory centre there.

The extreme pain that the patient had suffered from in the neck, especially on movement, was not due to the compression of the cord; for cord compression is painless if the posterior nerve roots are not irritated. It was due to simultaneous pressure exerted by the tumour upon the upper cervical nerve roots, particularly those of the left side.

This case is instructive in many ways; we would lay particular stress upon two of these.

First, we would emphasise the importance of paresis of the trunk muscles of one side as a point which is of the very greatest assistance in distinguishing a case of hemiplegia of spinal origin from one in which the lesion is higher up; this has been fully discussed above.

Secondly, this case indicates very clearly that when one side of the spinal cord is compressed sufficiently to cause hemiplegia, *the anæsthesia may be upon the same side as the paresis*. It is always a great difficulty with students, both in their physiological and in their clinical studies, to understand and believe the statement of the books that a unilateral lesion of the cord causes paralysis on the same side as the lesion and anæsthesia upon the opposite side. It always seems more probable upon *a priori* grounds that a unilateral cord lesion, if complete, will cause paresis and paræsthesia, both upon the same side as the lesion itself. The case described would have been more conclusive if the patient had happened to die when the paralysis was absolutely confined to the right side; but in her, as the paralysis spread, so did the anæsthesia; and the anæsthesia was in the same parts as the paralysis. That is what one would expect, though it is absolutely contrary to the teaching of the books. It is much to be desired that any reader who may have seen a similar case will not hesitate to record it, because such cases are not of everyday occurrence; and it is only by gathering them together that the question of which side of the body will be anæsthetic when there is a unilateral lesion of the cord can be definitely and finally settled. We venture the opinion that other cases will occur, as in the above, with anæsthesia upon the same side as the paralysis, both being upon the same side as the lesion in the cord.

